# Epidemiological profile of patients utilising public oral health services in Limpopo province, South Africa

**DOI:** 10.4102/phcfm.v9i1.1206

**Published:** 2017-07-12

**Authors:** Lawrence K. Thema, Shenuka Singh

**Affiliations:** 1 Discipline of Dentistry, School of Health Sciences, University of KwaZulu-Natal, South Africa; 2 Teaching and Learning, College of Health Sciences, University of KwaZulu-Natal, South Africa

## Abstract

**Background:**

Despite the impact of oral diseases on the quality of life, there is limited updated evidence on oral health status in Limpopo province.

**Objectives:**

To determine the epidemiological profile of patients utilising public oral health services in Limpopo province.

**Method:**

This was a descriptive retrospective clinical chart review conducted in five purposively selected district hospitals in Limpopo province. The collected data included the patient’s sociodemographic information, reasons for dental consultation, information on the dental or oral diseases and the treatment received. Five hundred clinical files were systematically selected (100 from each district hospital) for the period 01 January 1995 to 31 December 2013. Data were collected using the World Health Organization’s indicator age groups, namely 6-year-olds, 12-year-olds, 18-year-olds and 35–44-year-old groups. A data capturing sheet was used to record the collected information. Data were analysed using the statistical software package for social sciences SPSS version 23.0.

**Results:**

The majority of patients were in the age group of 6 to 20 years (*n* = 375, 75%). The majority were male patients (*n* = 309; 62%). Dental caries was the most common complaint (*n* = 298, 60%). The second most common main complaint in this age group was retained primary or deciduous teeth (*n* = 60, 12%) affecting children mainly in the age group of 6 to 12 years. The most common clinical procedure across all five districts was dental extractions (*n* = 324, 64%). Other clinical interventions included scaling and polishing (*n* = 33, 12%) and dental restorative care (*n* = 20, 3%).

**Conclusion:**

There is an urgent need to reorient oral health service delivery in Limpopo province to focus more on preventive oral health programmes.

## Background

Oral diseases remain a major public health concern in South Africa given their prevalence and impact on the quality of life.^1,2^ Despite these concerns, there is an unclear picture of the distribution of oral diseases across provinces and districts because of limited reliable epidemiological data and poor district oral health information systems.^3^ The South African National Children Oral Health Survey of 1999–2002 was one of the few national surveys to report on oral health status in South Africa, but this survey focused primarily on children aged 6, 12 and 15 years.^4^ This survey indicated that the highest prevalence of dental caries among children was recorded in the Western Cape where almost 80% of children needed oral health care and the lowest was recorded in Limpopo province (Table 1). The only reliable national data on adult oral health status were obtained in 1989, during the apartheid era.^2,5^ Apart from the sporadic and inconsistent epidemiological data on dental caries, periodontal diseases, malocclusion and dental fluorosis, there is very little recorded information on gingivitis, oral cancer, oral manifestation of HIV or AIDS and orofacial trauma.^6,7^

**TABLE 1:** Prevalence of dental caries and untreated caries by age group in South Africa.

Variables	Age group									
	4–5†		6†		6		12		15	
	Caries (%)	Untreated caries (%)	Caries (%)	Untreated caries (%)	Caries (%)	Untreated caries (%)	Caries (%)	Untreated caries (%)	Caries (%)	Untreated caries (%)
Weighted national mean	50.59	46.56	60.32	55.11	6.28	5.13	36.91	30.31	50.98	42.17
Provinces										
Western Cape	77.1	72.0	82.3	75.2	9.6	6.9	61.8	51.6	81.1	70.7
Northern Cape			84.1	82.7	16.4	14.8	47.3	44.2	62.8	55.2
Eastern Cape	58.9	53.7	67.7	63.6	4.1	3.7	49.0	32.7	63.8	48.4
Free State	60.1	57.8	59.2	56.8	4.9	4.0	36.8	33.3	54.5	50.6
KwaZulu-Natal	52.4	50.8	64.8	59.9	6.2	5.3	38.7	34.9	50.9	46.3
Gauteng	49.1	37.6	59.7	50.5	4.9	3.9	34.3	26.6	49.9	31.1
North West	41.0	39.5	52.3	48.2	7.8	7.4	27.5	25.0	39.0	35.5
Mpumalanga	40.2	35.1	56.2	48.4	10.1	4.8	29.7	26.6	41.4	36.8
Limpopo	31.3	30.8	37.2	33.8	4.9	4.8	15.8	14.1	28.4	24.1

*Source*: Department of Health. Report: National Children’s Oral Health Survey, South Africa. 1999–2002, 2003:9

† Primary dentition.

The aim of this study was to determine the epidemiological profile of patients utilising public oral health services in order to encourage a broader scope and reach of dental services in Limpopo province.

This study formed part of a larger research project examining oral health services in Limpopo province. Overall, oral health planning in Limpopo is somewhat constrained by limited reliable epidemiological data for planning and resource allocation. Statistics South Africa estimates a provincial population of about 5.3 million in five districts, namely Vhembe, Capricorn, Mopani, Sekhukhune and Waterberg, with almost 90% of the population living in non-urban areas. There are high levels of unemployment, poverty, poor sanitation and unsafe drinking water, coupled with the additional burden of malnutrition, unhealthy lifestyle practices and communicable diseases such as HIV or AIDS.^8^

## Methods

The study used a descriptive, retrospective clinical chart review design. The study aimed at identifying patients accessing oral health clinical care at the identified health facilities. Clinical records are an important source for information on disease profiles, communication between patient and service provider, and continuity of clinical care.^9^ Data were collected from patients’ clinical records at five purposively selected district hospitals in Limpopo province, namely Tshilidzini Hospital in Vhembe, St. Ritas Hospital in Sekhukhune, Letaba Hospital in Mopani, Mokopane Hospital in Waterberg and Zebediela Hospital in Capricorn district. These hospitals provide oral health and dental services to patients referred from primary health care clinics, community health care centres and other hospitals. Systematic random sampling was used to select 500 (*n* = 500) patient files. Data were collected in accordance with the World Health Organization indicator age groups, namely the 6-year-old, 12-year-old, 18-year-old and the 35–44-year-old groups.^10^ The available clinical files were selected by the appointed hospital administrative clerk (based on written reference to oral health management in the clinical notes) and handed over to the researcher. The administrative clerk was blinded to the objectives of the study so as to eliminate any potential bias in the selection of the patient clinical records. A pilot study was conducted using 10 patient clinical records to identify any challenges related to the file retrieval and data capturing processes. These records were not included in the final study sample. The researcher then identified every second clinical file from the available records for the study purpose. Twenty-five clinical files were selected for each indicator cohort group, adding up to a total of 100 files to represent each region. The available patient clinical files and records were collected and reviewed for the period 01 January 1995 to 31 December 2013. The study focused on clinical records after 01 January 1995, because health services became available without out-of-pocket payment from this period onwards. All issues of confidentiality and data security were maintained. The clinical files were not removed from the hospital premises.

A data capturing sheet was used to collect data on the patient’s age, level of education, residential district, employment status, home language and ability to pay for treatment. The second category of the data capturing sheet collected data on the patient’s dental history, clinical examination, duration or onset of the disease presentation, type of treatment done, length of period for the identified treatment regime and recording of other identified health conditions. A coding system was used to code collected data.

Data were entered into Microsoft Excel format, by regions and cohort age groups, thereby ensuring completeness of the data recorded. Data were analysed using the statistical software package for social sciences SPSS version 23.0. Univariate descriptive statistics such as frequency and mean distribution were conducted for all variables.

### Ethical consideration

Ethical clearance was provided by Biomedical Research Ethical Committee of University of Kwazulu-Natal (BREC REF: BE 327/14) and the Limpopo Department of Health (Ref 4/2/2).

## Results

The majority of patients presenting for oral health care at the identified health facilities were in the age group of 6 to 20 years (*n* = 375, 75%) (Table 2). Almost three-quarters of the study sample were male patients (*n* = 309; 62%). The most common home languages spoken by patients were Sepedi (56%), Xitsonga (20%), Tshivenda (18%), and English or Afrikaans (<3%).

**TABLE 2:** Age distribution of the study sample.

Age (years)	Capricorn		Mopani		Sekhukhune		Vhembe		Waterberg		Total (%)
	Male	Female	Male	Female	Male	Female	Male	Female	Male	Female	
6–10	16	9	17	8	17	8	13	12	15	10	125 (25.0)
11–15	15	10	18	7	16	9	16	9	16	9	125 (25.0)
16–20	15	10	16	9	13	12	14	11	14	11	125 (25.0)
31–35	5	4	6	3	6	4	7	2	6	3	46 (9.2)
36–40	6	5	2	5	5	2	6	3	7	4	45 (9.0)
41–45	3	2	5	4	6	2	4	3	4	1	34 (6.8)
**Total**	**60**	**40**	**64**	**36**	**63**	**37**	**60**	**40**	**62**	**38**	**500 (100)**

### Oral conditions

The majority of patients visited the dental clinic for relief of pain and sepsis. Dental caries was the most common main complaint (*n* = 298, 60%), and 80% of these patients were between 6 and 18 years old (Table 3). Only 10% of patients indicated the ability to pay for treatment. Retained primary or deciduous teeth were the second most common main complaint (*n* = 60, 12%) and presented mainly in the age group of 6 to 12 years.

**TABLE 3:** Oral conditions in the under-19 age groups.

Diseases	Age			Total (%)
	6 years	12 years	18 years	
Caries	70	81	88	239 (47.8)
Retained	34	23	3	60 (12.0)
Trauma	5	11	5	21 (4.2)
Sensitive	1	0	0	1 (0.2)
Pericoronitis	0	0	1	1 (0.2)
Cancer	0	1	2	3 (0.6)
Periodontal	0	2	18	20 (4.0)
Fluorosis	0	4	0	4 (0.8)
Impaction	0	0	1	1 (0.2)
Examination	15	2	5	22 (4.4)

Periodontal disease was identified as another main complaint (*n* = 57, 11%). A small number of patients sought treatment for dental trauma (*n* = 37, 7%), but 21 of these patients were under the age of 19 (*n* = 21, 4%). Dental fluorosis (*n* = 4, 0.8%) and pericoronitis (*n* = 3, 0.6%) were also recorded in the under-19 age group. A small number of patients (*n* = 28, 6%) attended the dental clinic for a routine dental check-up. Fifteen of these patients were in the 6 years age group (Table 3).

### Dental treatment services

The most common clinical procedure across all five districts was dental extractions (*n* = 324, 64%) (Table 4). Other clinical interventions such as scaling and polishing (cleaning of teeth) were performed on small number of patients (*n* = 33, 12%). Similarly, dental restorative care (fillings) was also performed on a relatively small number of patients (*n* = 20, 3%). Sutures are generally placed after a dental extraction and this procedure was recorded in 13 clinical files (4%).

**TABLE 4:** Dental service provision.

Treatment	Capricorn	Mopani	Sekhukhune	Vhembe	Waterberg	Total number (%)
Extraction	71	74	60	68	51	324 (64.3)
Scaling	2	12	10	0	9	33 (12.0)
Examination	3	0	0	26	0	29 (4.0)
Filling	0	6	5	0	9	20 (3.0)
Suturing	0	10	0	3	0	13 (3.6)
Medication	2	0	0	0	0	2 (6.4)
Other	-	-	-	-	-	6.7

## Discussion

The results of this study provide an interesting picture of the prevalence of oral diseases (from the profile of patients seeking dental services) in Limpopo province, its distribution across the age spectrum and the utilisation of dental services. Dental caries remains the most common oral disease in Limpopo province, and this observation is consistent with other findings on oral disease occurrence in South Africa and other parts of Africa.^3,7,11^ The results indicate that patients sought dental treatment primarily for the relief of pain and sepsis. This finding is also consistent with other studies that cite pain as the main reason for patients seeking dental treatment.^12,13^ The implication of these findings is that the prevalence of dental caries might actually be increasing in the under-19 age group and is contradictory to the results obtained in the South African National Children Oral Health Survey of 1999–2002. This observation has important implications for oral health planning in Limpopo province.

Although low levels of periodontal disease were recorded in the under-19 age group, this is a cause for concern. Adolescents can be at greater risk of periodontal disease because of exposure to factors such as ‘low socio-economic status, poor education, low dental care utilisation, poor oral hygiene levels, smoking, psychosocial stress and genetic factors’.^14^ More research is required to explore the possible reasons for periodontal disease occurrence in this age group.

### Dental utilisation rates

The results indicate that 62% of male patients sought dental treatment at the identified health facilities. This observation is consistent with the findings by Taiwo et al.^15^ that a greater proportion of men sought dental treatment. There is further supporting evidence in the literature that an association exists between dental anxiety (fear of dental treatment) and poor dental utilisation patterns, especially among women from low socioeconomic and disadvantaged backgrounds.^15,16,17^ Access to dental treatment could also be problematic because of the associated transport costs. Apart from dental caries, 12% of patients in this study sought dental treatment for retained primary or deciduous teeth. Murshid^18^ also observed in his study that almost 11% of children sought dental treatment for retained primary teeth. This is probably because of poor parental understanding of the tooth exfoliation process. The utilisation patterns of dental services can be attributed to a number of factors. These include unmet need and demands of patients that could be influenced by literacy levels, attitudes and perceptions towards oral health self-care, coupled by the availability of dental services.^15^ Patients tend to seek dental treatment mostly when pain or discomfort is experienced.^16^

The results further indicate that only a small number of restorations and preventive procedures such as scaling and polishing were recorded. The availability of dental services is dependent on adequate facilities, appropriately trained oral health workers, funding and resource allocation, and fully functioning dental units.^15,19^ The quality of dental services could be hampered if the appropriate staff is not available or there is lack of resources for the proper functioning of the dental clinics.^19^ Affordability of dental treatment must also be taken into account. Free dental services (without out-of-pocket payment) are only available in primary health care facilities and community health care centres. Patients are required to pay for dental treatment at hospital-based facilities (although at a minimal cost); therefore, services such as fillings, scaling and polishing could be perceived as non-essential because of the associated costs.^20^

Patients’ attitudes to and perceptions of oral health care, therefore, contribute to dental utilisation rates. Extraction of teeth could be considered a quick-fix solution to pain and discomfort without seeing the benefits of saving and restoring the dentition.^15,16,17,21^ Although the importance of the availability of resources cannot be overstated, dental professionals’ attitudes towards service provision must also be considered. The literature does suggest that in some developing countries, dental staff could be mostly engaged in dental extractions and this leaves very little time for restorative or preventive care.^21,22,23^

The results indicate a focus on facility-based clinical care with very little emphasis on preventive oral health care. Oral diseases such as dental caries are largely preventable, and the mechanisms to control and prevent its occurrence are simple and easy to implement.^1,2,6^ The national policies on oral health care in South Africa are very clear on the need for preventive care that focuses on empowering individuals and communities on oral health self-care practices (tooth brushing and dental flossing, access to additional fluoride uptake and reduction of sugar consumption). These strategies should form part of a comprehensive approach to address dental caries, including the prevalence of early childhood caries.^24^ This comprehensive approach should include all role players such as children, parents, caregivers, educators, oral health workers and other stakeholders.^25^

All of these factors necessitate a review of oral health planning and implementation in Limpopo province. More effort is required for a clear preventive strategy that is supported by funding, appropriate dental personnel and buy-in from relevant stakeholders. The current oral health promotion strategies that focus on primary schoolgoing children should be strengthened but these strategies need to be integrated into the wider community upliftment programmes (such as mother and child oral health promotion programmes), aimed at improving health and oral health outcomes.

### Limitations of the study

This study examined oral health service delivery utilisation rates over a period of 18 years. The study provided valuable insight into oral health service delivery in Limpopo province, specifically from a patient utilisation perspective. There were, however, challenges related to clinical records. These included missing records, difficulty in tracing the movement of files and lack of systems for tracking missing records and files. There were long-standing delays in tracking patient files from the archives and this was generally remedied through the creation of new patient files for the patient’s recall visits to the health institutions. This resulted in fragmented and inconsistent reference to the patient’s previous health records. There was inadequate recording, abbreviated recording and there were challenges in confirming the accuracy of the clinician’s ability to record the diagnosis and treatment plan. There was also no recorded information to indicate the number of patients that did not honour the referral process (patients who did not attend the referral hospital despite being referred for further clinical management).

Although, there are inevitable shortcomings, this study clearly highlights the need for oral health planning and prevention. The process of documenting and storing patient clinical records must also be reviewed.

## Conclusion

The results of the study indicate that dental extractions dominate the dental utilisation pattern in Limpopo province. There is an urgent need to reorient oral health service delivery to focus more on preventive oral health programmes. Such preventive programmes need to focus especially on children, parents or caregivers and teachers and should address children’s dietary intake (sugar consumption), tooth brushing methods and access to fluoridated toothpaste.

## References

[1] Naidoo S Oral health and nutrition for children under five years of age: A paediatric food-based dietary guideline. S Afr J Clin Nutr. 2013;26(3 Suppl):S150–S155.

[2] Singh S Dental caries rates in South Africa: Implications for oral health planning. S Afr J Epidemiol Infect. 2011;26(4 Pt II):259–261.

[3] Reddy M, Singh S Dental caries status in six-year-old children at health promoting schools in KwaZulu-Natal. S Afr Dent J. 2015;70(9):396–401.

[4] Department of Health Report: National Children’s Oral Health Survey, South Africa. 1999–2002; Pretoria: Department of Health; 2003; p. 9.

[5] Van Wyk PJ, Van Wyk C Oral health in South Africa. Int Dent J. 2004;54(6 Suppl 1):373–377. https://doi.org/10.1111/j.1875-595X.2004.tb00014.x 15631099 10.1111/j.1875-595x.2004.tb00014.x

[6] Petersen PE, Bourgeois D, Bratthall D Oral health information system – Towards measuring progress in oral health promotion and disease prevention. Bull World Health Organ. 2005;89(9):689–690. PMC2626332 16211160

[7] Thorpe S Oral health issues in the African regions: Current situation and future perspectives. American Dental Education Association. J Dent Educ. 2006;70(11) (8–15 Suppl):1–8.

[8] Strategic Planning Committee Annual performance plan 2013/14. Limpopo: Department of Health and Social Development; 2015.

[9] Rigby M, Georgiou A, Hyppönen H, et al Patient portals as a means of information and communication technology support to patient-centric care coordination – The missing evidence and the challenges of evaluation. Yearbook Med Inform. 2015;10(1):148–159. https://doi.org/10.15265/IY-2015-007 10.15265/IY-2015-007 PMC4587055 26123909

[10] World Health Organization Oral health surveys. Basic methods. 5th ed. Geneva: WHO Press; 2013; p. 14–15.

[11] Abid A, Maatouk F, Berrezouga L, et al Prevalence and severity of oral diseases in the Africa and middle east region. Adv Dent Res. 2015;27(1):10–17. https://doi.org/10.1177/0022034515582062 26101335 10.1177/0022034515582062

[12] Ogbebor OG, Azodo CC Reasons for seeking dental healthcare services in a Nigerian missionary hospital. Sahel Med J. 2016;19:38–43. https://doi.org/10.4103/1118-8561.181901

[13] Masiga MA Presenting chief complaints and clinical characteristics among patients attending the Department of Paediatric Dentistry Clinic at the University of Nairobi Dental Hospital. East Afr Med J. 2005;82(12):652–655. 16619711 10.4314/eamj.v82i12.9372

[14] Nanaiah KP, Nagarathna DV, Manjunath N Prevalence of periodontitis among the adolescents aged 15–18 years in Mangalore City: An epidemiological and microbiological study. J Indian Soc Periodontol. 2013;17(6):784–789. https://doi.org/10.4103/0972-124X.124507 24554891 10.4103/0972-124X.124507 PMC3917211

[15] Taiwo OA, Soyele OO, Ndubuizu GU Pattern of utilization of dental services at Federal Medical Centre, Katsina, Northwest Nigeria. Sahel Med J. 2014;17:108–111. https://doi.org/10.4103/1118-8561.140294

[16] Ajayi DM, Arigbede AO Barriers to oral health care utilization in Ibadan, South West Nigeria. Afr Health Sci. 2012;12:507–513. 23515140 10.4314/ahs.v12i4.17 PMC3598293

[17] Fotedar S, Sharma KR, Bhardwaj V, Sogi GM Barriers to the utilization of dental services in Shimla, India. Eur J Gen Dent. 2013;2:139–143. https://doi.org/10.4103/2278-9626.112314

[18] Murshid EZ Children’s ages and reasons for receiving their first dental visit in a Saudi community. Saudi Dent J. 2016;28:142–147. https://doi.org/10.1016/j.sdentj.2015.12.003 27656081 10.1016/j.sdentj.2015.12.003 PMC5021814

[19] Nyamuryekung’e KK, Lahti SM, Tuominen RJ The relative patient costs and availability of dental services, materials and equipment in public oral care facilities in Tanzania. BMC Oral Health. 2015;15:74 https://doi.org/10.1186/s12903-015-0061-3 26126654 10.1186/s12903-015-0061-3 PMC4487056

[20] Western Cape Government Free dental care for you and your family [homepage on the Internet]. 2015 [cited 2016 Oct 17]. Available from: https://www.westerncape.gov.za/general-publication/free-dental-care-you-and-your-family

[21] Mordohai N, Reshad M, Jivraj S, Chee W Factors that affect individual tooth prognosis and choices in contemporary treatment planning. Br Dent J. 2007;202:63–72. https://doi.org/10.1038/bdj.2007.23 17255985 10.1038/bdj.2007.23

[22] Anyanechi C, Chukwuneke F Survey of the reasons for dental extraction in eastern Nigeria. Ann Med Health Sci Res. 2012;2(2):129–133. https://doi.org/10.4103/2141-9248.105659 23440287 10.4103/2141-9248.105659 PMC3573506

[23] Bayat F, Murtomaa H, Vehkalahti MM, Tala H Does dental insurance make a difference in type of service received by Iranian dentate adults? Eur J Dent. 2011;5(1):68–76. 21311609 PMC3037191

[24] Kolisa Y Assessment of oral health promotion services offered as part of maternal and child health services in the Tshwane Health District, Pretoria, South Africa. Afr J Prim Health Care Fam Med. 2016;8(1):794 https://doi.org/10.4102/phcfm.v8i1.794 10.4102/phcfm.v8i1.794 PMC4845516 27247154

[25] Çolak H, Dülgergil ÇT, Dalli M, Hamidi MM Early childhood caries update: A review of causes, diagnoses, and treatments. J Nat Sci Biol Med. 2013;4(1):29–38. https://doi.org/10.4103/0976-9668.107257 23633832 10.4103/0976-9668.107257 PMC3633299

